# Preparing Austere Maritime Surgical Teams for Deployment During the COVID-19 Global Pandemic: Is It Time to Change the Training Pipeline?

**DOI:** 10.1093/milmed/usaa574

**Published:** 2021-01-05

**Authors:** Diego A Vicente, Obinna Ugochukwu, Michael G Johnston, Chad Craft, Virginia Damin, Matthew D Tadlock

**Affiliations:** Department of Surgery, Navy Medicine Readiness & Training Command, San Diego, CA, 92134, USA; Department of Surgery, Navy Medicine Readiness & Training Command, San Diego, CA, 92134, USA; Navy Medicine Readiness & Training Command, PSC 827 Box 1 FPO AE, Naples, 09617-0001, Italy; Navy Expeditionary Medical Training Institute, Camp Pendleton, CA, 92055-5223, USA; Navy Expeditionary Medical Training Institute, Camp Pendleton, CA, 92055-5223, USA; Department of Surgery, Navy Medicine Readiness & Training Command, San Diego, CA, 92134, USA

## Abstract

**Introduction:**

Navy Medicine's Role 2 Light Maneuver (R2LM) Emergency Resuscitative Surgical Systems (ERSS) are austere surgical teams manned, trained, and equipped to provide life-saving damage control resuscitation and surgery in any environment on land or sea. Given the restrictions related to the COVID-19 pandemic, the previously established pre-deployment training pipeline for was modified to prepare a new R2LM team augmenting a Role 1 shipboard medical department.

**Methods:**

The modified curriculum created in response to COVID-19 related restriction is compared and contrasted to the established pre-deployment R2LM ERSS curriculum. Subject Matter Experts and currently deployed R2LM members critically evaluate the two curricula.

**Results:**

Both curricula included the team R2LM platform training and exposure to cadaver based team trauma skills training. The modified curriculum included didactics on shipboard resuscitation, anesthesia and surgery, shipboard COVID-19 management, and prolonged field care in austere maritime environments.

**Conclusions:**

We describe Navy Medicines R2LM ERSS capability and compare and contrast the standard R2LM pre-COVID-19 curriculum to the modified curriculum. Central to both curricula, the standard R2LM platform training is important for developing and honing team dynamics, communication skills and fluid leadership; important for the successful function austere surgical teams. Several opportunities for improvement in the pre-deployment training were identified for R2LM teams augmenting shipboard Role 1 medical departments.

## INTRODUCTION

In general, the U.S. Navy utilizes four maritime surgical platforms including the hospital ship, aircraft carrier, fleet surgical teams (FST) embarked on amphibious assault ships, and the austere Role 2 Light Maneuver (R2LM) Emergency Resuscitative Surgical System (ERSS) platform. The R2LM/ERSS is designed to be scalable and highly mobile, enabling the team to embark on most surface ships within the fleet.^1^ The concept of augmenting Independent Duty Corpsman and ship’s corpsmen on a Role 1 maritime platform with a surgical capability is not a novel idea, as ships such as destroyers were augmented with surgical teams during World War II.^2^ This prior concept has been adapted and is currently being expanded in the modern U.S. Navy to provide resuscitative and surgical health service support during distributed maritime operations (DMO). These teams are typically composed of seven to nine critical wartime specialists drawn from various active duty and reserve commands and include a surgeon, anesthesia provider (anesthesiologist or certified nurse anesthetist), emergency physician, one to two critical care nurses, a respiratory technician, surgical technician, and/or physician assistant.^3,4^ Depending on the configuration of the R2LM package and available resources, the advertised capability includes performing damage-control resuscitation and damage-control surgery for up to six non-surgical and four surgical patients. The length of post-operative holding capability also depends on the type of package deployed but is typically limited to 24-48 hours.^3^

For the R2LM platform, pre-deployment training occurs as a team and includes the Navy Surgical Team Trauma and Advanced Resuscitation (NSTTAR) course at Navy Trauma Training Center (NTTC) and the R2LM course at Navy Expeditionary Medical Training Institute (NEMTI) in Southern California. This training is the same for active duty and reservist members of the R2LM team and allows members from separate commands to meet before deployment. In the current COVID-19 pandemic, various national and military precautionary measures and travel restrictions have been implemented to ensure a medically ready deployable force resulting in the need to alter various training pipelines. To adapt to this situation, we recently implemented an alternative curriculum to enhance the training of a recently deployed R2LM team augmenting a Role 1 shipboard medical department. We compare and contrast the standard and alternative R2LM curriculums, identify opportunities for improvement, and provide recommendations for future training.

## METHODS

In response to various restrictions related to the COVID-19 pandemic, the officer-in-charge of an R2LM team utilized a combination of resources and subject matter experts (SME) from NEMTI and Navy Medicine Readiness & Training Command (NMRTC) San Diego as well as leaders in trauma education from all three branches of service to develop a training curriculum focusing on austere maritime surgery. We evaluated the standard R2LM pre-COVID-19 curriculum and the alternative curriculum and identified the pros and cons of each. Finally, SME and currently deployed R2LM members critically evaluated the existing trainings for gaps relative to the expected level of performance.

## RESULTS

The components of the established pre-COVID-19 curriculum are listed in Table I and are comprised of two separate training courses: NSTTAR and R2LM platform training. The NSTTAR course at NTTC is an 11-day classroom, mannequin- and cadaver-based curriculum comprised of standard off-the-shelf courses routinely used by all of the services for pre-deployment training of austere surgical teams including the Emergency War Surgery Course (EWSC), Fundamentals of Critical Care Support, and the 1-day American College of Surgeons Advanced Surgical Skills for Exposure in Trauma (ASSET) as well as some exposure to care of the injured military working dog, resuscitative endovascular balloon occlusion of the aorta (REBOA), walking blood bank (WBB) skills, and other expeditionary skills (Table I). The curriculum is capped off with a half-day sequential team summative simulation experience utilizing a unique perfused fresh human cadaver model where surgical teams manage complex traumatic injuries in simulated bleeding cadavers in real time.^5^

**TABLE I.** Standard R2LM ERSS Pre-Deployment Training

NSTTAR	R2LM platform training
Knowledge and skills exposure Care of the military working dogs REBOA simulator Austere orthopedics, neurosurgery, and obstetrics Intraosseus access Walking blood bank	Develops and refines team dynamics Open and effective communication Team trust, empowerment, and accountability Dynamic subordination/ fluid leadership Defines roles and shared responsibilities
Standardized off-the-shelf curricula Emergency war surgery course Fundamentals of critical care support ASSET (1-day course)	Tactical planning Concept of operations Medical planning R2LM gear familiarization Daily SME team evaluation and feedback
Unique half-day perfused fresh human cadaver sequential team summative simulation experience	High-fidelity trauma simulation in diverse austere expeditionary environments Hasty field conditions Buildings of opportunity Shipboard environment utilizing NEMTI-adapted DDG ship simulator. Night operations utilizing night vision goggles. V-22 Osprey simulator Walking blood bank exposure

Abbreviations: ASSET, Advanced Surgical Skills for Exposure in Trauma; ERSS, Emergency Resuscitative Surgical Systems; NEMTI, Navy Expeditionary Medical Training Institute; NSTTAR, Navy Surgical Team Trauma and Advanced Resuscitation; R2LM, Role 2 Light Maneuver; REBOA, resuscitative endovascular occlusion of the aorta; SME, subject matter expert.

The characteristics of R2LM platform-specific training are also listed in Table I. The training provides high-fidelity trauma simulation utilizing perfused surgical cut suits in a diverse expeditionary environment designed to develop and refine team dynamics including the honing of communication skills, trust, empowerment, and accountability designed to create a culture of dynamic subordination and fluid leadership such that the teams function as a single organism during high-stress austere conditions. This team building is particularly important for R2LM as these teams are composed of individuals from separate commands with a diverse set of R2LM-relevant backgrounds and experience. Other highlights of this training include R2LM authorized medical allowance list gear familiarization, tactical planning, night operations, and training in the shipboard, land, and air simulators. Notably, the training is designed to prepare teams to perform trauma care in hasty field conditions and buildings of opportunity.

The characteristics of the alternative curriculum are listed in Table II. Although the R2LM team could not travel to NTTC, NSTTAR instructors met the team at NEMTI and delivered an abbreviated 2-day NSTTAR course. This consisted largely of didactics, with only limited practical experience with mannequins and integrated team training. The team subsequently completed the described R2LM platform training (Table I) and accomplished the goals of building team structure and communication. In order to enhance the hands-on experience and surgical training, impromptu arrangements were made for five of the seven members of the R2LM team to attend additional in-person training at (NMRTC) San Diego. The remaining two members attended didactic training virtually but were unable to participate in the hands-on training. The primary focus of this 2-week training was the new 2-day ASSET+ perfused cadaver-based in-depth knowledge and skills training in vascular exposures for trauma, as well as training in the technical basics of emergency obstetrics and orthopedics. Additionally, ASSET+ includes a summative skills assessment for the surgical team participating. This training element was critical for the surgeon, PA (Physician Assistant), and scrub tech to gain comfort and confidence in working together to deliver coordinated complex surgical care. In addition to ASSET+, daily SME lectures and hands-on experience were arranged in the bioskills lab with cadaver and mannequin models to include WBB and REBOA curricula. Other topics covered included vascular access, interosseous access, shipboard surgery and resuscitation, shipboard COVID-19 management, and prolonged field care.

**TABLE II.** Alternative R2LM ERSS Pre-Deployment Training

Abbreviated 2-day NSTTAR didactics
Standard R2LM platform training (Table I)
ASSET+ (2-day course) ASSET+ cadaver-based curriculum Formal summative skills assessment Knowledge and skills exposure to austere orthopedics, neurosurgery, and obstetrics
In-depth knowledge and skills training REBOA Vascular and intraosseus access Formal walking blood bank curriculum Shipboard COVID-19 management
Austere maritime-specific R2LM didactics Shipboard triage, resuscitation, and surgery Shipboard ERSS configurations Anesthesia care Lessons learned
Austere R2LM didactics Prolonged casualty care Operational critical care Special operations surgical team-lessons learned

Abbreviations: ASSET, Advanced Surgical Skills for Exposure in Trauma; NSTTAR, Navy Surgical Team Trauma and Advanced Resuscitation; R2LM, Role Two Light Maneuver; REBOA, Resuscitative Endovascular Occlusion of the Aorta.

## DISCUSSION

We describe the development of a modified R2LM pre-deployment training program that was implemented in response to various restrictions and safety concerns related to the global COVID-19 pandemic. Although objective training outcomes data were not available, in this Brief Report we consider the benefits and deficits of the characteristics of the established pre-COVID-19 R2LM training and the alternative pre-deployment R2LM training by the currently deployed R2LM team and ERSS SMEs. These considerations have yielded several opportunities for improvement. This report also highlights some of the pre-deployment training challenges surgical teams are facing preparing for deployments during the COVID-19 pandemic.

The primary benefit of both training pipelines is the incorporation of the R2LM platform training and consequent development of team confidence and communication. The clinical and tactical decision-making skills that the scenarios provide are consistently lauded by students, particularly in terms of honing constructive communication and team dynamics in a high-stakes environment. The incorporation of cadaver-based training to develop technical comfort with complex trauma procedures is also a major benefit of both pipelines. In contrast to the established training, however, the modified training does not include the summative team experience with a perfused cadaver at NTTC.^5^ This deficit was partially covered in the modified curriculum with the ASSET+ course and summative team drills at R2LM platform training. Of note, the Defense Health Agency (DHA) is in the process of replacing the EWSC with the ASSET+ curriculum and its summative skills assessment, which will be required training for certain critical war specialties every 2-3 years.

The major deficit from the established pre-COVID-19 training was the lack of shipboard-specific training. This is a vital training element to prepare a maritime surgical team, and SME lectures from prior ERSS “lessons learned” significantly influenced resuscitative room setup and the team structure for the currently deployed R2LM team. The major deficit of both training pipelines as noted by the currently deployed R2LM team, and multiple prior R2LM after action reports, is the lack of real-world clinical experience with trauma, burn, and critical care patients before deployment. Additional noted deficits include the development of shipboard protocols for controlled substances and blood products as well as the development of supply chains in austere environments. This latter portion cannot be understated given that R2LM teams are currently deployed without dedicated administrative and logistical support for re-supply.

Finally, a major deficit of both training pipelines is a strategy to prevent skills degradation while on deployment. As the duration of shipboard R2LM deployments is extended from the previously established days or weeks to potentially several months on expeditionary sea-base platforms, mid-deployment trauma training should be considered to mitigate degradation of clinical skills experienced during prolonged deployments without clinical volume.

Based on the authors’ experiences with R2LM and ERSS platforms and experience with both the established and alternative training pipelines, we developed a list of recommendations (Table III) to improve the R2LM/ERSS training pipeline. These recommendations are primarily relevant for R2LM surgical teams augmenting Role 1 medical departments on surface vessels, and improved training pipelines will become particularly important as the Navy expands these teams for DMO. The importance of these R2LM teams was highlighted by Navy Surgeon General Vice Admiral Faison during his 2018 testimony to the Subcommittee on Defense of the Senate Committee on Appropriations as part of Navy Medicine’s modernization efforts to provide scalable and adaptable expeditionary medical capabilities. With this in mind, the Defense Health Program Fiscal Year 2021 President’s Budget provides for annual training of nine seven-member R2LM teams^6^ with the goal of establishing multiple active R2LM platforms to reduce the time and distance to life saving damage-control resuscitation and surgery to amphibious and afloat conventional or special operation forces during DMO.^1,6^

**TABLE III.** Recommendations for Future Maritime Austere Surgical Teams

Recommendations: Decentralize the current curriculum at NTTC and partner with NMRTC’s in San Diego, Camp Lejeune, Portsmouth, and Jacksonville such that ASSET+, FCCS, and formal REBOA training are taught where large numbers of Navy Medicine’s forward deployed caregivers are stationed to expand the availability of this important training. Refocus the NTTC curriculum to concentrate on clinical skills sustainment experiences in trauma, burn, and critical care, utilizing a team-based approach. Incorporate maritime-specific R2LM didactics emphasizing lessons learned across various maritime platforms including shipboard triage, resuscitation, anesthesia, surgery, and various R2LM/ERSS configurations that have been successful in past operations. Include guidance on navigating administrative obstacles such as the supply chain, patient movement, and blood product procurement. Incorporate prolonged casualty care curriculum including the management of burns, smoke inhalation, drowning, hypothermia, and operational critical care. Incorporate historical lessons learned from combat and non-combat maritime mass casualty incidents. Incorporate formal REBOA training course, beyond current introductory exposure to all forward deployed surgical teams including ideal indications, associated complications, and complication management. Incorporate formal WBB curriculum beyond exposure into pre-deployment training. Implement WBB capability (policy, training, materiel, and pre-screening ship’s crew) across all Role 1 surface medical departments that may be augmented by an R2LM surgical team. Continue current Navy and Defense Health Agency efforts to develop regional skills-sustainment partnerships. Develop training to establish shipboard protocols for controlled substances and blood product management as well as training on establishing supply chains.

Abbreviations: ASSET, Advanced Surgical Skills for Exposure in Trauma; ERSS, Emergency Resuscitative Surgical Systems; FCCS, Fundamentals of Critical Care Support; NMRTC, Navy Medicine Readiness & Training Command; NTTC, Navy Trauma Training Center; R2LM, Role 2 Light Maneuver; REBOA, resuscitative endovascular balloon occlusion of the aorta; WBB, walking blood bank.

Although the operational concept is still being developed, during both contested and non-contested DMO, individual components of the naval force will be more dispersed, and these forces will be connected by a “comprehensive operational architecture” of new and developing technology to provide sea control over a larger geographic area^7,8^ Therefore, Role 2 afloat surgical teams supporting DMO will likely have to provide prolonged care of injured and acutely ill patients in austere maritime environments.

This model is in contrast to the previously established operational paradigms of rapid transfer from the ship to a higher level of care and has been well documented in four major incidents over the past 30 years. In 1987, for example, a missile-attack on the USS STARK (FFG 31) resulted in 21 casualties, which were transported off the ship within 2-3 hours of injury. Similarly, the 37 casualties from the suicide terrorist bombing of the USS COLE (DDG 67) were transported to local hospitals within 1.5 hours of the attack.^9,10^ During the more recent 2017 collisions of the USS FITZGERALD (DDG 62) and the USS McCAIN (DDG 56), casualties were evacuated off the ship within 8 hours.^11,12^ The documented injuries from these incidents were severe and included burns, smoke inhalation, aspiration of sea water or fuel, immersion, hypothermia, and injuries resulting from blunt force or blast mechanisms of injury including traumatic brain injury, rib, facial and long bone fracture, and solid organ and traumatic injuries requiring blood transfusion.^9–13^ These injuries rapidly overwhelmed the available shipboard Role 1 medical assets and, hence, necessitated rapid transfer of casualties.

Of note, the recent mass casualty incident in 2017 on USS BATAAN (LHD 5) provides a contemporary example of maritime combat casualty care in a non-contested DMO environment. At the time of the incident, there were three surgical teams embarked on the *BATAAN*, including an FST) an R2LM/ERSS team and a Surgical Resuscitation Team. After a Blackhawk helicopter crash, six injured patients were rapidly transferred via MV-22 Osprey (with a surgical team aboard) to the USS BATAAN where the three surgical teams and the ship’s medical department successfully delivered damage-control resuscitation and surgery followed by prolonged casualty care for six critically ill casualties requiring a total of 55 units of blood products. Because of their relative geographic isolation, casualties remained on board for 38-42 hours before transfer to a Role 4 level of care.^14,15^

Although the management of this incident was a success with three well-trained surgical teams on-board the Casualty Receiving and Treatment Ship, it does prompt the question of how a single seven-person R2LM team with the described lack of clinical trauma training would manage casualties from a missile attack, collision or helicopter crash-related mass casualty incident and care for those patients for several days before MEDEVAC. This is unquestionably a setup for preventable loss of life. Therefore, clinical training and real-world experience in trauma, burn management, smoke inhalation, neuro-critical care, operational critical care, and prolonged casualty care are mandatory for optimal patient outcomes.

Beyond the described training deficits, the R2LM teams augmenting Role 1 surface fleet medical assets have limited available blood products and limited number of patients from whom to draw low titer group O blood for a WBB. Over the last 20 years of war in Iraq and Afghanistan, Military Medicine has re-learned an important lesson from World War II: a true surgical capability does not exist without appropriate resuscitation.^16^ In modern warfare, this includes the availability of either cold-stored low titer group O whole blood (LTOWB), fresh LTOWB from a WBB, or approximating fresh whole blood from component therapy.^17,18^

Our recommendations address many of these opportunities for improvement including the need for appropriate resuscitation (blood) to have a true surgical capability (Table III). Additionally, our modified curriculum addresses many lessons learned that R2LM teams may encounter across various surface platforms including where on the ship to set up the resuscitation and operating areas, where to hold critically ill patients and the types of injuries that may be encountered in the distributed maritime environment, and emphasizes the principles or prolonged casualty care. The need for prolonged casualty care training has been recognized across all of the services and is currently being studied by a Tactical Combat Casualty Care working group and NEMTI R2LM platform training has begun to incorporate aspects of the described maritime-specific curriculum (Table II). Additionally, the lessons learned from our and others experience are currently in the process of being reviewed and reported through Navy Medicine’s Operational Clinical Community in order implement doctrinal change. Although not the focus of this report, future implementation of these recommendations into R2LM training will require objective data to gauge the efficacy of these trainings and provide real-time feedback on future improvements.

The current NSTTAR curriculum (Table I) is designed for all naval austere surgical platforms including those teams assigned to the U.S. Marine Corps, FST, and all R2LM/ERSS platforms potentially creating a training bottleneck. With the travel restrictions and other precautions related to the current COVID-19 pandemic, this training bottleneck has been exacerbated. Not only will decentralizing the current curriculum increase access to this important training, it will minimize cross-country travel during the current pandemic. Additionally, decentralized training could be extended to other Role 2 surgical assets and will decrease unnecessary time away from home for forward deployed caregivers, likely improving professional satisfaction and retention. This will also allow NTTC to prioritize training for OCONUS providers who may not have access to decentralized training and to refocus the curriculum to concentrate on clinical care using a team-based model. Finally, neither of the pre-deployment training pipelines described provide R2LM team members any real-world clinical experience caring for actual trauma, burn, or critically ill patients. Decentralizing the curriculum will allow the embedded cadre to focus on developing clinical skills sustainment opportunities in trauma, burn, and critical care for forward deployed caregivers. Beyond the pre-deployment setting, an expanded decentralized curriculum could be considered for teams at risk for critical resuscitation skills deterioration in setting of prolonged deployments with low clinical volume.

This report has several inherent limitations. It is a descriptive comparison of two different training pipelines without any reportable objective outcomes data. A further prospective comparison of the two pre-deployment training programs is needed looking at defined outcomes such as individual provider and team knowledge and confidence before and after each training pipeline as well as time to team task completion for each progressive scenario. As much of the R2LM training occurs at night during hasty field conditions, objective data can be difficult to obtain. However, given these limitations, this report highlights an unprecedented problem in providing adequate pre-deployment training to surgical teams during the current COVID-19 pandemic. However, through this process several opportunities for curriculum improvement were identified and are in the process of being implemented.

The lack of complex trauma and non-trauma patient care opportunities is a universal problem across the Military Health System, which is beyond the scope of this manuscript. At this time, there are four military treatment facilities (Naval Medical Center Camp Lejeune, Brooke Army Medical Center-San Antonio, William Beaumont Army Medical Center, Madigan Army Medical Center) that provide critical trauma care support to their surrounding civilian communities through either state designation or American College of Surgeons Committee on Trauma accreditation. However, the Lejeune (level 3) and San Antonio (level 1) have the most robust civilian trauma volume. Although trauma center accreditation may not be feasible at every military treatment facility, each service along with the DHA is working to implement policy to correct the known gap in trauma experience by developing partnerships with high-volume academic centers. While needed, as these partnerships are developed and implemented, the priority should be to develop skills sustainment opportunities that are in close proximity to large populations of forward deployed caregivers, to minimize time away from home in addition to deployments and other required training activities.

## CONCLUSION

Adapting to unprecedented circumstances in the setting of COVID-19, the authors developed a modified R2LM curriculum to enhance pre-deployment training for the described austere maritime surgical platform. In the process, we identified several opportunities for improvement, and put forth recommendations to ensure that these teams are ready to deploy, providing a damage-control resuscitation and surgery capability augmenting Role 1 shipboard medical departments during DMO.
